# Supplementary material for: Multi-Stage Transcriptome Analysis Revealed the Growth Mechanism of Feathers and Hair Follicles during Induction Molting by Fasting in the Late Stage of Egg Laying
Source: Biology (Basel). 2023 Oct 19;12(10):1345. doi: 10.3390/biology12101345 (PMC10603888; doi:10.3390/biology12101345)
Supplement: Supplementary file 1 [file biology-12-01345-s001.zip › biology-2592238-supplementary.pdf]

Table S1. RNA-Seq quality control result.

Sample	RawDatas	CleanData(%)	Adapter(%)	LowQuality(%)	Q20(%)	Q30(%)	N(%)	GC(%)
**F0-1**	37922294	37586978 (99.12%)	40028 (0.11%)	295288 (0.78%)	97.45%	92.99%	0.00%	48.49%
**F0-2**	42962174	42641178 (99.25%)	23932 (0.06%)	292010 (0.68%)	97.66%	93.50%	0.00%	48.35%
**F0-3**	51184048	50824572 (99.30%)	26030 (0.05%)	327088 (0.64%)	97.87%	94.01%	0.00%	48.78%
**F15-1**	48341388	47952960 (99.20%)	28676 (0.06%)	353896 (0.73%)	97.52%	93.16%	0.00%	49.01%
**F15-2**	51604866	51238376 (99.29%)	26912 (0.05%)	333560 (0.65%)	97.78%	93.73%	0.00%	48.79%
**F15-3**	45635536	45310194 (99.29%)	28458 (0.06%)	291612 (0.64%)	97.86%	93.92%	0.00%	48.92%
**R5-1**	47764930	47431546 (99.30%)	25252 (0.05%)	302504 (0.63%)	97.74%	93.67%	0.00%	48.88%
**R5-2**	53110862	52726336 (99.28%)	32776 (0.06%)	345214 (0.65%)	97.70%	93.59%	0.00%	49.19%
**R5-3**	44682424	44371746 (99.30%)	26324 (0.06%)	279064 (0.62%)	97.65%	93.47%	0.00%	49.28%
**R32-1**	44375150	44076610 (99.33%)	26426 (0.06%)	266204 (0.60%)	97.60%	93.30%	0.00%	49.42%
**R32-2**	40707226	40320306 (99.05%)	47662 (0.12%)	338672 (0.83%)	97.37%	92.88%	0.00%	49.56%
**R32-3**	37860616	37498374 (99.04%)	43930 (0.12%)	317818 (0.84%)	97.50%	93.22%	0.00%	50.53%

Table S2. Results table of different genes for each group (first 10 rows).

Group	ID	Symbol	log2(FC)	*P*-Value	FDR
F0-VS-F15	MSTRG.13499	ZP1	-5.5502	1.25E-27	2.05E-23
	ENSGALG00000017394	INSIG1	-1.3555	6.75E-19	5.54E-15
	ENSGALG00000029308	PNPLA3	-1.8128	3.68E-14	2.01E-10
	ENSGALG00000001331	ST14	-1.6602	2.47E-13	1.01E-09
	ENSGALG00000048920	--	-4.5634	7.39E-13	2.43E-09
	ENSGALG00000001857	C1QTNF12	-2.9977	8.17E-11	2.23E-07
	ENSGALG00000003078	MMD	-1.7249	3.29E-10	6.97E-07
	ENSGALG00000052022	--	-3.9734	3.40E-10	6.97E-07
	ENSGALG00000041491	ACKR4	-2.0856	1.01E-09	1.85E-06
	MSTRG.14534	--	-1.4638	1.50E-09	2.46E-06
	ENSGALG00000012790	DSP	0.714813076	0.004742914	0.120668547
	ENSGALG00000000608	CDH1	1.536775734	0.008355285	0.158073707
	ENSGALG00000000299	PKP1	0.869955313	0.059873646	0.380035396
F0-VS-R5	ENSGALG00000005541	GJB1	-4.17094	1.72E-68	2.84E-64
	ENSGALG00000004519	TRPV2	-1.84631	3.13E-47	2.58E-43
	MSTRG.13499	ZP1	-5.76074	1.50E-35	8.22E-32
	ENSGALG00000010476	BNIP3	-1.48534	4.20E-29	1.73E-25
	MSTRG.14534	--	-1.79749	1.38E-27	4.55E-24
	ENSGALG00000041257	ACVR1C	-2.42159	1.84E-26	5.05E-23

	ENSGALG00000002421	FAM213A	-1.94159	1.26E-24	2.97E-21
	ENSGALG00000005553	NLGN3	-1.42384	2.55E-23	5.26E-20
	ENSGALG00000009700	PDK4	-4.23715	3.87E-22	7.08E-19
	MSTRG.8206	--	-1.45148	5.37E-21	8.84E-18
	ENSGALG00000012790	DSP	1.134127581	0.002999347	0.050846954
	ENSGALG00000000608	CDH1	1.462441393	0.00656066	0.084789455
	ENSGALG00000000299	PKP1	1.522322649	0.001920788	0.037769743
F0-VS-R32	ENSGALG00000009724	GREM1	-3.29616	1.24E-20	2.03E-16
	ENSGALG00000013057	USP18	-1.98614	1.24E-19	1.02E-15
	ENSGALG00000012873	SERPINB5	1.859907	1.00E-17	5.47E-14
	ENSGALG00000010866	AREG	1.998151	6.21E-17	2.55E-13
	ENSGALG00000017040	C4	-2.01371	1.07E-16	3.51E-13
	ENSGALG00000051882	MUC4	5.077869	4.73E-16	1.29E-12
	ENSGALG00000015345	CRYBG1	2.668477	5.74E-16	1.34E-12
	ENSGALG00000054252	TM4SF1	5.933619	2.88E-15	5.92E-12
	ENSGALG00000037011	CILP2	3.085276	7.31E-14	1.33E-10
	ENSGALG00000046632	--	1.751577	1.04E-13	1.71E-10
	ENSGALG00000012790	DSP	2.312326731	5.08E-12	4.63E-09
	ENSGALG00000000608	CDH1	2.485873645	1.18E-07	1.98E-05
	ENSGALG00000000299	PKP1	2.430335865	5.12E-08	1.02E-05
F15-VS-R5	ENSGALG00000029308	PNPLA3	2.811057	4.08E-42	6.73E-38
	ENSGALG00000007114	APOA1	-2.77026	7.27E-26	5.99E-22
	ENSGALG00000048409	TXNIP	-1.56208	3.10E-19	1.71E-15
	ENSGALG00000049450	IGLC1	2.718289	5.90E-16	2.43E-12
	ENSGALG00000041926	HEXIM1	-1.31622	2.12E-15	7.01E-12
	ENSGALG00000037943	PRKCB	1.941684	1.19E-14	3.26E-11
	ENSGALG00000048920	--	5.024988	1.75E-14	4.13E-11
	ENSGALG00000052022	--	4.498031	6.75E-14	1.39E-10
	ENSGALG00000012006	RTN1	-1.17164	1.52E-13	2.78E-10
	ENSGALG00000050515	IGHA1	2.462235	2.90E-13	4.78E-10
	ENSGALG00000000299	PKP1	0.652367336	0.133604024	0.522936233
F15-VS-R32	ENSGALG00000055004	--	6.83919	3.37E-35	5.54E-31
	ENSGALG00000004804	TGM3	4.889517	1.90E-24	1.57E-20
	ENSGALG00000054020	--	9.650214	1.29E-23	7.07E-20
	MSTRG.13499	ZP1	4.798641	2.21E-22	9.10E-19
	ENSGALG00000025872	TGM6	3.470953	4.24E-20	1.40E-16
	MSTRG.8963	--	5.979878	2.00E-19	5.47E-16
	MSTRG.8962	--	5.93466	3.76E-19	8.84E-16
	ENSGALG00000050822	--	3.646558	1.08E-17	2.21E-14
	ENSGALG00000048920	--	5.323816	5.20E-16	9.51E-13
	ENSGALG00000044307	PLA2G4E	4.627306	1.12E-15	1.84E-12

	ENSGALG00000012790	DSP	1.597513655	1.71E-06	0.000370254
	ENSGALG00000039985CDH1	CDH1	-0.659903042	0.010198072	0.190429606
	ENSGALG00000000299	PKP1	1.560380552	0.000101556	0.009030825
R5-VS-R32	MSTRG.13499	ZP1	5.009159	5.54E-28	9.14E-24
	ENSGALG00000007511	ITGB2	-1.71765	2.27E-24	1.87E-20
	ENSGALG00000035818	--	5.931713	5.61E-24	3.09E-20
	ENSGALG00000027874	CHAC1	3.892139	1.21E-20	4.98E-17
	ENSGALG00000043909	--	-5.32043	3.98E-20	1.31E-16
	ENSGALG00000042148	FKBP5	2.490048	4.17E-19	1.15E-15
	ENSGALG00000015419	PENK	-4.26115	3.57E-16	8.41E-13
	ENSGALG00000050893	CCR2	-1.95589	5.83E-15	1.20E-11
	ENSGALG00000001571	MYO1F	-1.64806	8.17E-15	1.50E-11
	ENSGALG00000005725	CSF1R	-1.22459	1.13E-13	1.77E-10
	ENSGALG00000012790	DSP	1.17819915	0.001457466	0.041279949
	ENSGALG00000000608	CDH1	1.023432251	0.018019582	0.224257868
	ENSGALG00000000299	PKP1	0.908013217	0.022645743	0.256449977

Note: in the table, the *DSP*, *CDH1*, and *PKP1* are placed, along with the first ten genes in each group.
